# Copresence of *tet*(K) and *tet*(M) in Livestock-Associated Methicillin-Resistant Staphylococcus aureus Clonal Complex 398 Is Associated with Increased Fitness during Exposure to Sublethal Concentrations of Tetracycline

**DOI:** 10.1128/AAC.00426-16

**Published:** 2016-06-20

**Authors:** Jesper Larsen, Julie Clasen, Julie E. Hansen, Wilhelm Paulander, Andreas Petersen, Anders R. Larsen, Dorte Frees

**Affiliations:** a Department of Microbiology and Infection Control, Statens Serum Institut, Copenhagen, Denmark; b Department of Veterinary Disease Biology, Faculty of Health and Medical Sciences, University of Copenhagen, Frederiksberg C, Denmark; c European Programme for Public Health Microbiology, European Centre for Disease Prevention and Control, Solna, Sweden

## Abstract

The tetracycline resistance gene *tet*(K) was shown to be integrated within the predominant staphylococcal cassette chromosome *mec* (SCC*mec*) element of Danish livestock-associated methicillin-resistant Staphylococcus aureus CC398 (LA-MRSA CC398). These LA-MRSA CC398 isolates already possessed *tet*(M), but the acquisition of *tet*(K) significantly improved their fitness at sublethal concentrations of tetracycline. Because *tet*(K) is genetically linked to SCC*mec*, the use of tetracycline in food animals may have contributed to the successful spread of LA-MRSA CC398.

## TEXT

Livestock-associated methicillin-resistant Staphylococcus aureus CC398 (LA-MRSA CC398) has become a major public health concern as a result of its rapid, uncontrolled spread in food animals and farm workers, from which it spills over into the surrounding community (1–3). The emergence of LA-MRSA CC398 has been linked to the intensive use of antimicrobial drugs in food animals (4, 5). Tetracycline is one of the most commonly used antibiotic classes in food animals (6). In some LA-MRSA CC398 isolates, the tetracycline resistance plasmid pT181 is found integrated between the *ccr* region and the J1 region within the type Vc staphylococcal cassette chromosome *mec* (SCC*mec*) element (7). The plasmid is flanked by IS*257* (also known as IS*431*) and appears to have been integrated into SCC*mec* Vc as a consequence of IS*257* insertion between the replication initiation gene, *repC*, and *tet*(K), which encodes an efflux pump conferring tetracycline resistance (7, 8). However, it remains unclear whether acquisition of *tet*(K) plays a role in the coselection of methicillin resistance, given that virtually all LA-MRSA CC398 isolates are already resistant to tetracycline due to the presence of another tetracycline resistance gene, *tet*(M), encoding a so-called ribosomal protection protein, which reduces the affinity of ribosomes for tetracycline when GTP is present (4, 9–11). To gain insights into the role of *tet*(K) in LA-MRSA CC398, we investigated the prevalence, genetic organization, and fitness effect of *tet*(K) during *in vitro* exposure to tetracycline.

**FIG 1:**
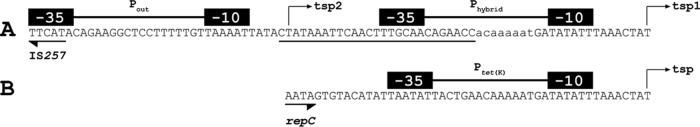
Organization of *tet*(K) promoters. (A) Integrated and free forms of pT181 in SCC*mec* Vc-bearing isolates. (B) Free form of pT181 in isolates harboring nonsubtypeable SCC*mec* V elements. The solid boxes represent −10 and −35 sequences of the promoters P_hybrid_, P_out_, and P_*tet*(K)_. The arrows above the sequences represent transcription start points. The arrows underneath the sequences represent the start of IS*257* and the end of *repC*, respectively. The imperfect 27-bp terminal inverted repeat bounding IS*257* is underlined. Lowercase letters represent the 8-bp target duplication. See reference 13 for further details.

The Danish LA-MRSA CC398 collection used in this study included 146 human isolates collected by Statens Serum Institut between 2004 and 2009; the isolates had been characterized previously for *spa* type, presence of *tet*(M), and antimicrobial susceptibilities in a different study by Larsen and colleagues, who investigated the epidemiology of LA-MRSA CC398 in humans in Denmark from 1999 to 2011 (3). The presence of *tet*(M) and *tet*(K) was investigated using a PCR assay described elsewhere (12), and the results showed that *tet*(M) and *tet*(K) were present in 144 (99%) and 89 (61%) of the 146 LA-MRSA CC398 isolates, respectively. Of note, the two *tet*(M)-negative isolates were positive for *tet*(K). SCC*mec* typing (see methodological details in the supplemental material) showed that 104 isolates carried SCC*mec* Vc (71%). SCC*mec* IVa was found in 12 isolates, and SCC*mec* Vb was found in 14 isolates, while we detected nonsubtypeable SCC*mec* V elements in 11 isolates and a type VII-like SCC*mec* element in 5 isolates. The *tet*(K) gene was strongly associated with the predominant SCC*mec* Vc element; of the 89 *tet*(K)-bearing isolates, 86 also carried SCC*mec* Vc, while 3 isolates harbored nonsubtypeable SCC*mec* V elements. In other words, SCC*mec* Vc-bearing isolates carried the *tet*(K) gene at a much higher frequency than isolates harboring other types of SCC*mec* elements (83% [86 of 104 isolates] versus 7% [3 of 42 isolates]). Two in-house PCR assays were used to assess whether pT181 was present in the free form (PCR 1) or integrated into SCC*mec* (PCR 2). Primers were designed on the basis of the nucleotide sequence of SCC*mec* Vc in LA-MRSA CC398 strain JCSC6944 (DDBJ/EMBL/GenBank accession no. AB505629) (see Fig. S1 and Table S1 in the supplemental material). PCR 2 produced an amplicon with the expected size (1,119 bp) in all 86 *tet*(K)- and SCC*mec* Vc-bearing isolates, supporting their possession of the IS*257*-flanked integrated form of pT181 between the *ccr* region and the J1 region, which is similar to the genetic organization in strain JCSC6944 (7). Interestingly, PCR 1 resulted in a 1,235-bp amplicon in all 86 isolates, suggesting that they also harbor the free form of pT181, in which IS*257* is inserted between *repC* and *tet*(K). The presence of IS*257* upstream of *tet*(K) is known to confer higher levels of tetracycline resistance and better fitness during exposure to tetracycline due to enhanced *tet*(K) transcription from the IS*257*-derived hybrid promoter, which is stronger than the native *tet*(K) promoter (13). Sanger sequencing of the PCR 1 and PCR 2 products from a representative *tet*(K)- and SCC*mec* Vc-bearing LA-MRSA CC398 isolate revealed that both the integrated form and free form of pT181 contained the IS*257*-derived hybrid promoter (Fig. 1). These results suggest that *tet*(K) transcription is enhanced in these isolates independent of pT181 integration. In contrast, only the free form of pT181 was identified in the 3 LA-MRSA CC398 isolates harboring nonsubtypeable SCC*mec* V elements. The resulting PCR 1 amplicons were shorter than expected (438 bp), and Sanger sequencing of a representative isolate revealed that this pT181 variant did not harbor the IS*257* insertion between *repC* and *tet*(K), indicating that *tet*(K) transcription is directed by the weaker native *tet*(K) promoter in these isolates (Fig. 1).

The tetracycline MIC was determined for all LA-MRSA CC398 isolates by use of the broth microdilution method, according to European Committee on Antimicrobial Susceptibility Testing guidelines (14, 15). Tetracycline MIC data were transformed to the natural log to approximate a normal distribution prior to statistical analysis. The geometric mean tetracycline MIC was shown to be significantly higher for isolates carrying both *tet*(M) and *tet*(K) genes compared with those carrying only *tet*(M) (106 versus 58 mg/liter; *P* < 0.0001), using the unpaired two-tailed Student's *t* test (GraphPad Prism software, version 5.0; GraphPad Software, Inc., San Diego, CA). The tetracycline MICs for the three isolates harboring nonsubtypeable SCC*mec* V elements were relatively low (64 mg/liter) compared with other isolates carrying both *tet*(M) and *tet*(K), which may be due to the fact that they possessed the pT181 variant with the weaker native *tet*(K) promoter. The tetracycline MICs for the two isolates carrying only *tet*(K) were 64 and 128 mg/liter.

**FIG 2:**
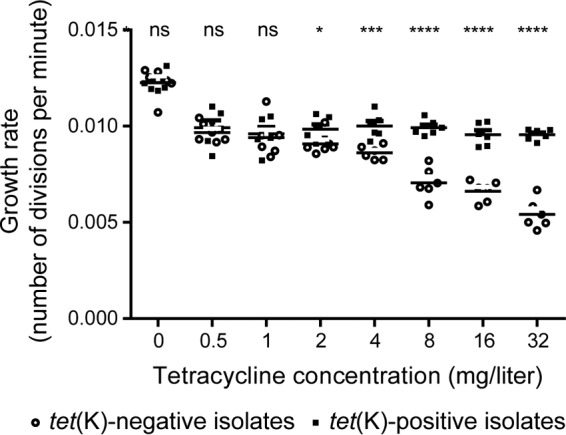
Comparison of the growth rates of six *tet*(K)-positive and six *tet*(K)-negative LA-MRSA CC398 isolates. The isolates are described in Table S1 in the supplemental material. Single cultures of each isolate were grown overnight in MHB, diluted to an optical density at 600 nm of 0.07 in 200 μl fresh MHB containing 0, 0.5, 1, 2, 4, 8, 16, or 32 mg/liter tetracycline, and grown at 37°C in a PowerWave 340 reader (BioTek, Winooski, VT). The OD_600_ was measured over time and used to calculate the number of divisions per minute during exponential growth. The statistical differences between the growth rates were determined using the unpaired two-tailed Student's *t* test (GraphPad Prism software version 5.0; GraphPad Software, Inc., San Diego, CA). Abbreviations: ns, not significant (*P* > 0.05); *, *P* ≤ 0.05; **, *P* ≤ 0.01; ***, *P* ≤ 0.001; ****, *P* ≤ 0.0001.

LA-MRSA CC398 may become exposed to different concentrations of tetracycline, depending on where the bacteria are present (e.g., in different food animals and host tissues or in the farm environment) as well as on the dose, the administration route (e.g., intravenously or via feed or drinking water), and the pharmacokinetic and pharmacodynamics properties of the drug. To test whether the presence of *tet*(K) also conferred a fitness advantage during exposure to sublethal concentrations of tetracycline, we compared the exponential growth rates of six *tet*(K)-positive and six *tet*(K)-negative isolates representing the predominant LA-MRSA CC398 genotype and antibiotic resistance profiles (see Table S2 in the supplemental material). In brief, single cultures of each isolate were incubated overnight in Mueller-Hinton broth (MHB), diluted to an optical density at 600 nm (OD_600_) of 0.07 in 200 μl fresh MHB containing 0 to 32 mg/liter tetracycline, and grown at 37°C in a PowerWave 340 reader (BioTek, Winooski, VT). The OD_600_ was measured every 15 s for 4.5 h and was used to calculate the number of divisions per minute during exponential growth. While the growth rate of *tet*(K)-negative isolates decreased progressively with increasing tetracycline concentrations, *tet*(K)-positive isolates continued to grow at a constant rate; in fact, *tet*(K)-positive isolates were shown to have a significantly higher growth rate at ≥2 mg/liter, using the unpaired two-tailed Student's *t* test (GraphPad Prism software, version 5.0; GraphPad Software, Inc., San Diego, CA) (Fig. 2).

Taken together, these results indicate that *tet*(K) confers an additional fitness advantage to *tet*(M)-bearing LA-MRSA CC398 in the presence of tetracycline, even at very low levels. Because *tet*(K) is genetically linked to SCC*mec* Vc, it is possible that the use of tetracycline in food animals has contributed to the successful spread of SCC*mec* Vc-bearing LA-MRSA CC398. Of note, SCC*mec* Vc also harbors the cadmium and zinc resistance gene, *czrC*, in the J1 region (7), and the use of zinc in pigs has been shown previously to coselect for methicillin resistance (16, 17). Future studies should assess whether the presence of the IS*257*-derived hybrid promoter in SCC*mec* Vc-bearing LA-MRSA CC398 leads to enhanced *tet*(K) transcription, higher levels of tetracycline resistance, and better fitness at different tetracycline concentrations and whether reduced use of tetracycline and zinc in food animals will lead to a reduction in the frequency of SCC*mec* Vc-bearing LA-MRSA CC398 in the animal population and in humans. The Ministry of Environment and Food of Denmark's initiative to reduce the overall use of antibiotics and metals in pigs, initiated in April 2015, is an important step in assessing the causal relationship between these drugs and methicillin resistance (18).

## Supplementary Material

Supplemental material
