# Supplementary material for: Copresence of tet(K) and tet(M) in Livestock-Associated Methicillin-Resistant Staphylococcus aureus Clonal Complex 398 Is Associated with Increased Fitness during Exposure to Sublethal Concentrations of Tetracycline
Source: Antimicrob Agents Chemother. 2016 Jun 20;60(7):4401–3. doi: 10.1128/AAC.00426-16 (PMC4914685; doi:10.1128/AAC.00426-16)
Supplement: Supplemental material [file supp_60_7_4401__index.html]

Copresence of tet(K) and tet(M) in Livestock-Associated Methicillin-Resistant Staphylococcus aureus Clonal Complex 398 Is Associated with Increased Fitness during Exposure to Sublethal Concentrations of Tetracycline — Supplemental material 

# Copresence of *tet*(K) and *tet*(M) in Livestock-Associated Methicillin-Resistant Staphylococcus aureus Clonal Complex 398 Is Associated with Increased Fitness during Exposure to Sublethal Concentrations of Tetracycline

## Supplemental material

- Supplemental file 1 -

  Additional experimental details and Supplemental Figure S1 and Tables S1 and S2.

  PDF, 44K
