# Supplementary material for: Copresence of tet(K) and tet(M) in Livestock-Associated Methicillin-Resistant Staphylococcus aureus Clonal Complex 398 Is Associated with Increased Fitness during Exposure to Sublethal Concentrations of Tetracycline
Source: Antimicrob Agents Chemother. 2016 Jun 20;60(7):4401–3. doi: 10.1128/AAC.00426-16 (PMC4914685; doi:10.1128/AAC.00426-16)
Supplement: Supplemental material [file AAC.00426-16_zac007165333so1.pdf]

SCC*mec* typing

The structural features unique to each of the type 1-5 *ccr* gene complexes and class A, B, and C2 *mec* gene complexes of the SCC*mec* element were determined by multiplex PCR assays described by Kondo et al. (1). The class C1 and C1.2 *mec* gene complexes were determined using a PCR assay and primers described by Ruppe et al. (2); the sizes of the PCR products estimated from the nucleotide sequences are 1,266 and 306 bp for *mecA*-IS*431*, in agreement with the fact that IS*431* is inserted 968 bp and 17 bp downstream of the *mecRI* start codon in *mec* gene complex C1 and C1.2, respectively (3,4). Subtyping of type IV (a-d) and type V (a-b) SCC*mec* was performed using multiplex PCR assays and primers that have been described elsewhere (1,5). The *czrC* gene, an indicator of type Vc SCC*mec* (4), was detected using a PCR assay and primers described by Cavaco et al. (6). SCC*mec* nomenclature was as proposed by the International Working Group on the Classification of Staphylococcal Cassette Chromosome Elements (7). For brevity, the type is indicated by Roman numerals and the subtype by a lower-case Latin letter. The *ccr* and *mec* gene complexes are indicated by an Arabic number and Latin letter, respectively, in parentheses. The MRSA strains COL (SCC*mec* I [1B]), N315 (SCC*mec* II [2A]), 85/2082 (SCC*mec* III [3A]), CA05 (SCC*mec* IVa [2B]), 8/6-3P (SCC*mec* IVb [2B]), 81/108 (SCC*mec* IVc [2B]), JCSC4469 (SCC*mec* IVd [2B]), WIS (SCC*mec* Va [5C2]), 50148 (SCC*mec* Vb [5C2&5]), 55488 (SCC*mec* Vc [5C2&5]), HDE288 (SCC*mec* VI [4B]), JCSC6082 (SCC*mec* VII [5C1]), and JCSC6945 (SCC*mec* X [6C1.2]) were used as controls for the PCR assays.

References

1. **Kondo Y, Ito T, Ma XX, Watanabe S, Kreiswirth BN, Etienne J, Hiramatsu K.** 2007. Combination of multiplex PCRs for staphylococcal cassette chromosome *mec* type assignment: rapid identification system for mec, ccr, and major differences in junkyard regions. Antimicrob Agents Chemother **51:**264-274.
2. **Ruppé E, Barbier F, Mesli Y, Maiga A, Cojocaru R, Benkhalfat M, Benchouk S, Hassaine H, Maiga I, Diallo A, Koumaré AK, Ouattara K, Soumaré S, Dufourcq JB, Nareth C, Sarthou JL, Andremont A, Ruimy R.** 2009. Diversity of staphylococcal cassette chromosome *mec* structures in methicillin-resistant *Staphylococcus epidermidis* and *Staphylococcus haemolyticus* strains among outpatients from four countries. Antimicrob Agents Chemother **53:**442-449.
3. **Berglund C, Ito T, Ikeda M, Ma XX, Söderquist B, Hiramatsu K.** 2008. Novel type of staphylococcal cassette chromosome *mec* in a methicillin-resistant *Staphylococcus aureus* strain isolated in Sweden. Antimicrob Agents Chemother **52:**3512-3516.
4. **Li S, Skov RL, Han X, Larsen AR, Larsen J, Sørum M, Wulf M, Voss A, Hiramatsu K, Ito T.** 2011. Novel types of staphylococcal cassette chromosome *mec* elements identified in clonal complex 398 methicillin-resistant *Staphylococcus aureus* strains. Antimicrob Agents Chemother **55:**3046-3050.
5. **Higuchi W, Takano T, Teng LJ, Yamamoto T.** 2008. Structure and specific detection of staphylococcal cassette chromosome *mec* type VII. Biochem Biophys Res Commun **377:**752-756.
6. **Cavaco LM, Hasman H, Stegger M, Andersen PS, Skov R, Fluit AC, Ito T, Aarestrup FM.** 2010. Cloning and occurrence of *czrC*, a gene conferring cadmium and zinc resistance in methicillin-resistant *Staphylococcus aureus* CC398 isolates. Antimicrob Agents Chemother **54:**3605-3608.
7. **International Working Group on the Classification of Staphylococcal Cassette Chromosome Elements (IWG-SCC).** 2009. Classification of staphylococcal cassette chromosome *mec* (SCC*mec*): guidelines for reporting novel SCC*mec* elements. Antimicrob Agents Chemother **53:**4961-4967.
8. **Larsen J, Petersen A, Sørum M, Stegger M, van Alphen L, Valentiner-Branth P, Knudsen LK, Larsen LS, Feingold B, Price LB, Andersen PS, Larsen AR, Skov RL.** Meticillin-resistant *Staphylococcus aureus* CC398 is an increasing cause of disease in people with no livestock contact in Denmark, 1999 to 2011. 2015. Euro Surveill **20:**pii=30021.

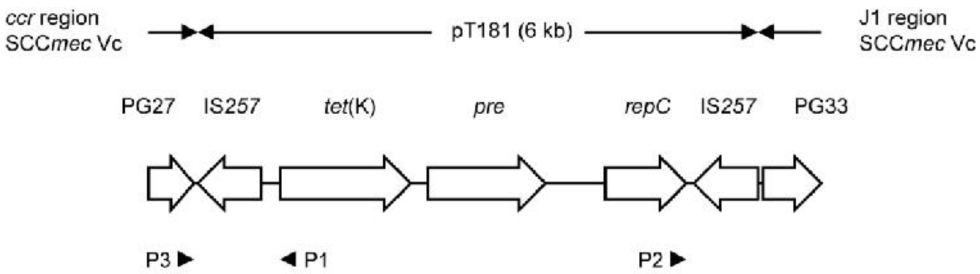

FIG. S1. Genetic organization of pT181 in LA-MRSA CC398 strain JCSC6944 (DDBJ/EMBL/GenBank accession no. AB505629). Black arrowheads indicate primer binding sites.

TABLE S1. Primers used to detect the two forms of pT181[a]

Primer	Nucleotide sequence (5´→3´)	Constructed on	Form detected	Expected size of product (bp)
PCR 1				
P1	TGCAGTGTTTACCCAGTTTGT	*tet*(K) (PG29)	pT181 in the free form	1,235
P2	GCCAGTCGATTTAACGGACT	*repC* (PG31)		
PCR 2				
P3	AAATCCGGCAACAAATGAAG	J1 region (PG27)	pT181 integrated into IS*257* within the J1 region of the type Vc SCC*mec* element	1,119
P1	TGCAGTGTTTACCCAGTTTGT	*tet*(K) (PG29)		

[a] Primers were designed on the basis of the nucleotide sequence of the type Vc SCC*mec* element in LA-MRSA CC398 strain JCSC6944 (DDBJ/EMBL/GenBank accession no. AB505629).

Table S2. SCC*mec* Vc-bearing LA-MRSA CC398 isolates used in the growth rate experiments [a]

Isolate	*spa* type	SCC*mec* type[b]	Resistance profile[c]	*tet*(M)	*tet*(K)	TET MIC (mg/L)[c]
A	t034	Vc	TET	+	-	32
B	t034	Vc	TET	+	-	32
C	t034	Vc	TET	+	+	128
D	t034	Vc	TET	+	+	128
E	t034	Vc	TET, CLI, ERY	+	-	32
F	t034	Vc	TET, CLI, ERY	+	-	64
G	t034	Vc	TET, CLI, ERY	+	+	128
H	t034	Vc	TET, CLI, ERY	+	+	128
I	t034	Vc	TET, CLI, ERY, STR	+	-	64
J	t034	Vc	TET, CLI, ERY, STR	+	-	64
K	t034	Vc	TET, CLI, ERY, STR	+	+	128
L	t034	Vc	TET, CLI, ERY, STR	+	+	128

[a] The isolates represent the predominant *spa* type-SCC*mec* combination, accounting for 58% (85/146) of the LA-MRSA CC398 isolates during 2004-2009 (8). Most of these 85 isolates exhibited one of three major antibiotic resistance profiles: tetracycline (24%); tetracycline, clindamycin, and erythromycin (15%); or tetracycline, clindamycin, erythromycin, and streptomycin (14%) (8).

[b] SCC*mec*, staphylococcal cassette chromosome *mec*.

[c] TET, tetracycline; CLI, clindamycin; ERY, erythromycin; STR, streptomycin.
